# Carbon‐Based Materials for Articular Tissue Engineering: From Innovative Scaffolding Materials toward Engineered Living Carbon

**DOI:** 10.1002/adhm.202101834

**Published:** 2021-10-12

**Authors:** Monsur Islam, Andrés Díaz Lantada, Dario Mager, Jan G. Korvink

**Affiliations:** ^1^ Karlsruhe Institute of Technology Institute of Microstructure Technology Hermann‐von‐Helmholtz‐Platz 1 Eggenstein‐Leopoldshafen 76344 Germany; ^2^ Department of Mechanical Engineering Universidad Politécnica de Madrid José Gutiérrez Abascal 2 Madrid 28006 Spain

**Keywords:** bones, carbon materials, carbon scaffolds, engineered living materials, osteochondral repair, tissue engineering

## Abstract

Carbon materials constitute a growing family of high‐performance materials immersed in ongoing scientific technological revolutions. Their biochemical properties are interesting for a wide set of healthcare applications and their biomechanical performance, which can be modulated to mimic most human tissues, make them remarkable candidates for tissue repair and regeneration, especially for articular problems and osteochondral defects involving diverse tissues with very different morphologies and properties. However, more systematic approaches to the engineering design of carbon‐based cell niches and scaffolds are needed and relevant challenges should still be overcome through extensive and collaborative research. In consequence, this study presents a comprehensive description of carbon materials and an explanation of their benefits for regenerative medicine, focusing on their rising impact in the area of osteochondral and articular repair and regeneration. Once the state‐of‐the‐art is illustrated, innovative design and fabrication strategies for artificially recreating the cellular microenvironment within complex articular structures are discussed. Together with these modern design and fabrication approaches, current challenges, and research trends for reaching patients and creating social and economic impacts are examined. In a closing perspective, the engineering of living carbon materials is also presented for the first time and the related fundamental breakthroughs ahead are clarified.

## Graphical abstract

Carbon‐based solutions in the area of osteochondral and articular repair and regeneration are emphasized by comprehensively reviewing the application of different carbon allotropes and the fabrication strategies employed to achieve the carbon scaffolds. Different challenges of the carbon‐based scaffolds are discussed toward clinical applications. A futuristic vision for engineering of carbon‐based living materials is laid out as a promising future direction.

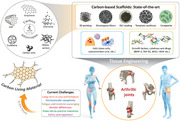

## 1 Introduction

Tissue engineering, regenerative medicine, and biofabrication pursue an ambitious objective of reconstructing damaged tissues by providing cells with the adequate biomimetic micro‐ and macro‐environment to deploy their healing power and, thereby, replacing traditional passive implants by active living biomaterials and structures. To this end, an increased number of combinations of geometries, materials, and technologies have been researched and developed during the last three decades, which has resulted in a myriad of synthetic cell niches, extracellular matrices, and tissue engineering scaffolds.^[^ ^1^, ^2^ ^]^

Despite these advances, most of the combinations are still being tested in laboratory environments and thus far from reaching patients, especially in those applications dealing with simultaneous repair of various interconnected tissues. Within these more demanding areas, the relevance of articular problems cannot be neglected. Their prevalence has importantly increased over the past decades due to combined factors like increased life expectancy, generalized sedentarism, aggressive sports practice, to cite a few. For an illustrative example, 25% of the population may develop symptomatic hip osteoarthritis during their lifetime.^[^ ^3^, ^4^ ^]^

Nowadays, articular restorations still rely on traditional metallic, ceramic, and polymeric implants, or employ autografts, allografts, and xenografts. While conventional implants are suboptimal in biomechanical performance, grafts can be a source of negative immune responses, all of which limit the long‐term viability of available solutions. Tissue engineering is emerging as the suitable choice in the near future. However, new material families, processing techniques, and manufacturing technologies should be studied to overcome current biomechanical mismatches, size‐related limitations, biocompatibility issues, and design‐controlled spatiotemporal tunability to the articular requirements and the actual healing processes.^[^ ^5^, ^6^ ^]^

Carbon and carbon‐based materials are often presented as the materials of the future, due to their special mechanical, electrical, thermal, tribological, and biological properties, which lead to high‐performance applications in all conceivable industrial fields, from biotechnology to aerospace, and from energy to electronics. Since the dawn of tissue engineering, carbon materials have been applied to musculoskeletal repair. Normally, a single type of carbon material has been employed for restoring one specific tissue, with different success rates. Interestingly, contemporary revolutions, like the discoveries of carbon nanotubes, fullerenes, and graphene, and continued advances in the synthesis, processing, and application of carbon‐based materials, have expanded the horizons of this family of materials, leading to the development of innovative biomedical devices and tissue engineering solutions.

The carbon‐based family provides an extremely versatile portfolio of biomaterials, with mechanical properties covering the whole spectrum of interest for interacting with the human body and with options for biomechanically replacing all kinds of articular tissues. In comparison to other synthetic biomaterials for replacing articular tissues, carbon‐based materials minimize mechanical mismatches and complement with interesting biocompatibility. From the authors' perspective, the potential of carbon‐based materials for repairing and regenerating human tissues is enormous, especially for treating osteochondral and articular defects. However, more systematic approaches to the engineering design of carbon‐based cell niches and scaffolds are needed, and extensive research should still address relevant challenges.

Our review focuses on the use of carbon‐based materials for osteochondral regeneration and articular tissue engineering, advancing on the previous studies that have treated bone,^[^ ^7^, ^8^ ^]^ cartilage,^[^ ^9^ ^]^ or ligament^[^ ^10^ ^]^ repair independently, or have concentrated on a single type of carbon material.^[^ ^11^, ^12^, ^13^ ^]^ In this study, all carbon materials are analyzed with a clear emphasis on achieving multi‐scale and multi‐material living constructs, as required for solving the more demanding tissue engineering challenges, like osteochondral and articular repair and regeneration, which involve several types of cells and tissues, with properties spanning over orders of magnitude.

The study begins with a comprehensive description of carbon materials and with an explanation of their benefits for regenerative medicine, highlighting their rising impact in the area of osteochondral and articular repair and regeneration. Consequently, the state‐of‐the‐art in carbon‐based materials for tissue engineering is presented, covering the applications of essential carbon materials including carbon nanotubes, graphene and graphene‐oxide, carbon dots, carbon fibers and meshes, glassy carbon, nano‐diamonds, and diamond‐like carbon.

Once the state‐of‐the‐art is illustrated, more innovative design and fabrication strategies for artificially recreating the cellular microenvironment within complex articular structures are discussed. Among them, the use of polymer–carbon and ceramic–carbon nanocomposites, pyrolysis of naturally occurring cellular precursors and additively manufactured carbon precursors, 3D printing of graphene foams, coating of carbon nanomaterials, textile‐based methods, and creation of composite structures by 3D printing with carbon filling are detailed.

Together with these modern design and fabrication approaches, current challenges and research trends for reaching patients and creating social and economic impacts are examined. Aspects which are considered here include: long‐term in vivo performance of carbon‐based solutions based on their fatigue behavior and material scavenging; reconstruction of microvascular complexity in carbon‐based solutions, a “holy grail” in tissue engineering; the path from laboratory test to industrialized production and mass customization; and the key safety and regulatory issues.

**Figure 1:**
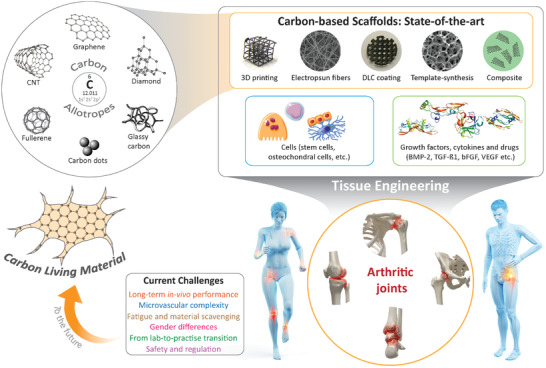
Graphical illustration depicting scope of the review paper.

Toward the future, the engineering of living carbon materials is introduced, to the authors' best knowledge for the first time, and the fundamental breakthroughs ahead are clarified. These include a shift to self‐assembly processes to achieve large‐scale defect reconstructions; promotion of smart responses to reach self‐sensing, shape‐morphing, and autonomously operating carbon materials and structures; and the accomplishment of self‐healing properties, possibly based on a self‐sufficient production of the living carbon materials of the next decades. In the authors' opinion, all this will reshape the fields of tissue engineering, regenerative medicine, and biofabrication in the years to come. The scope and structure of the review are schematically depicted in **Figure** **1**.

## 2 Why is Carbon Suitable for Tissue Engineering and Regenerative Medicine?

### 2.1 Carbon Allotropes

Carbon is a fascinating member of nature's material library. At ground state, carbon has an electronic structure of 1s^2^2s^2^2p^2^ with four electron vacancies in its outer electron shell. Such electronic arrangement allows carbon atoms to participate in forming robust covalent bonds with other carbon atoms in various hybridization states (sp, sp^2^, sp^3^), enabling the existence of several carbon allotropes in the solid state.^[^ ^14^, ^15^ ^]^ Among all the carbon allotropes, diamond and graphite are the only naturally occurring allotropes.^[^ ^16^ ^]^ Even though diamond and graphite are made up of only carbon atoms, their properties are very different. For example, diamond is the hardest material known and electrically an insulator. In comparison, graphite is soft and features a remarkable electrical conductivity.^[^ ^15^ ^]^ The reason for such contrasting properties between the two allotropes lies in their atomic arrangement.

#### 2.1.1 Diamond

Diamond features a face‐centered cubic (fcc) lattice structure, where sp^3^ hybridized carbon atoms form a tetrahedral configuration. One carbon atom located at the center of the tetrahedron establishes carbon‐carbon covalent bonds with four other carbon atoms at the four vertices of the tetrahedron (as shown in the carbon allotropes section of Figure 1). At each bond, the participating electron pair from two carbon atoms occupies different spin states, satisfying the Pauli exclusion principle, which renders the carbon‐carbon bonds extremely strong.^[^ ^17^ ^]^ Such strong carbon–carbon bonds are responsible for the excellent mechanical hardness and extremely poor electrical conductivity of diamond.

#### 2.1.2 Graphene, Graphene Oxide, and Reduced Graphene Oxide

**Figure 2:**
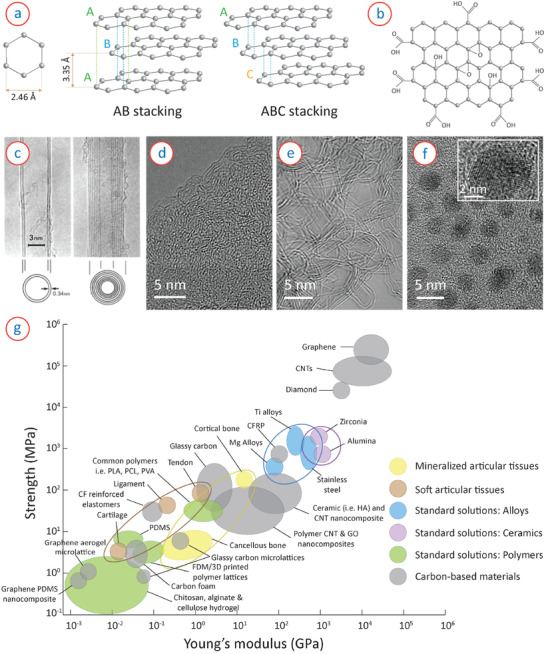
a) Lattice parameters and arrangement of graphene stacking in multi‐layer graphene. b) Surface groups attached to the graphene sheets in graphene oxide. c) TEM image of CNTs, showing tubular structure of MWCNTs. Reproduced with permission.^[^ ^32^ ^]^Copyright 1991, Springer Nature. d) TEM image of amorphous carbon, showing disordered and short‐range order in the microstructure. e) TEM micrograph of glassy carbon, showing turbostratic arrangement of long‐range graphitic planes. Reproduced with permission.^[^ ^41^ ^]^ Copyright 2018, Springer Nature. f) TEM image of carbon quantum dots, showing nanometric particle size. Inset shows crystalline arrangement of multiple graphene planes in an individual CQD. Reproduced with permission.^[^ ^43^ ^]^ Copyright 2019, Springer Nature. g) Comparison of strength and Young's modulus of carbon materials, illustrating the bio‐mechanical versatility of the carbon‐based solutions in comparison to other standard solutions.

The unit cell of graphite is called graphene, which was first isolated from bulk graphite by Andre Geim and Konstantin Novoselov in 2004.^[^ ^18^ ^]^ Graphene is characterized by a 2D sheet of sp^2^‐hybridized carbon atoms, where each carbon atom forms in‐plane covalent σ bonds with three other carbon atoms, forming planar arrays of 2D hexagonal lattice units (see “carbon allotropes” section of Figure 1), with a lattice constant of 2.46 Å. The in‐plane covalent σ bond results in a short inter‐atomic distance of ≈1.4 Å, which is significantly stronger than the sp^3^ bonds of diamond.^[^ ^19^ ^]^ Graphene can be categorized based on the number of layers: monolayer graphene, bi or double‐layer graphene and few‐layer graphene (2⩽layer⩽10).^[^ ^20^ ^]^ For bi‐layer and few‐layer graphene, the 2p_*z*_ orbitals of the carbon atoms, which are perpendicular to the planar structure, overlap with the 2p_*z*_ orbitals of the carbon atoms from the adjacent parallel graphene sheets, forming out‐of‐plane π bonds. The inter‐layer distance of graphene sheets in graphite is 3.35 Å. The graphene layers can be stacked in two different arrangements: AA stacking and AB stacking.^[^ ^21^ ^]^ In the AA configuration, all the carbon atoms are located vertically above each other, whereas AB stacking is characterized by two contiguous layers moved a distance of half of the lattice vector in the layer plane (**Figure** **2**a). AB stacking is the most energetically stable configuration of bi‐layer and few‐layer graphene.^[^ ^22^ ^]^ Few‐layer graphene, as well as graphite, features also rhombohedral ABC stacking (Figure 2a), where half of the carbon atoms of a third layer C vertically align with the carbon atoms in layer A, and the other half align with the carbon atoms in layer B.^[^ ^22^, ^23^ ^]^ It should be mentioned here that few‐layer graphene behaves very differently from bulk graphite.

When graphene layers are treated with oxidizing agents, polar groups are introduced to the graphene surface by widening the inter‐layer distance of graphene layers. Such modified graphene is called graphene oxide (GO). According to the L‐K model of GO, hydroxyl and ether groups are randomly distributed over the graphene surface, whereas the edge of the graphene layer features carboxyl and carbonyl groups (Figure 2b).^[^ ^24^ ^]^ GO contains both aromatic sp^2^ domains and aliphatic sp^3^ domains, which further facilitate surface interactions.^[^ ^25^ ^]^ The degree of aromatic and aliphatic carbon atoms depends on the degree of oxidation and distribution of the oxide groups over the graphene layer. Such modification of graphene layers leads to different surface properties for GO.^[^ ^25^, ^26^ ^]^ For example, GO is hydrophilic, whereas pristine graphene is hydrophobic. However, the oxygen containing groups results in a significant reduction in the electrical conductivity of GO.^[^ ^27^ ^]^ To regain the electrical conductivity, GO is typically converted to reduced graphene oxide (rGO), where GO is processed in various methods, including chemical, thermal, and electrochemical, to minimize the amount of oxygen‐containing groups.^[^ ^27^, ^28^ ^]^ rGO further features superior thermal and mechanical properties compared to GO, but still less than graphene due to the presence of higher surface defects.

#### 2.1.3 Fullerene

Fullerene is one of the first carbon allotropes discovered beyond graphite and diamond. Fullerene is found as spherical structures of carbon atoms in a Buckminster–Fuller spaceframe‐like arrangement. The atoms forming the spherical structure are sp^2^ hybridized, and each atom forms covalent bonds with three other adjacent carbon atoms, resulting in the formation of hexagonal and pentagonal configurations. These form a steady spherical shape. C_60_ (also known as Buckminsterfullerene) is the most commonly studied fullerene, where 60 carbon atoms form an icosahedron sphere with 12 pentagons and 20 hexagons.^[^ ^16^, ^29^ ^]^ The radius of C_60_ fullerene is 0.35 nm. The smallest fullerene structure that has been reported to date is C_20_, and theoretically, all fullerenes with C_20 + 2*F*_ (where *F*≠1 and *F*⩾0) structure are feasible.^[^ ^30^, ^31^ ^]^ The radius (*R*) of a fullerene structure (*C*_*n*_) can be estimated from Equation (1).^[^ ^29^ ^]^

(1) $$R=\frac{\sqrt{n}}{22}$$

#### 2.1.4 Carbon Nanotube

Carbon Nanotubes (CNTs) have been extensively investigated in various fields of research since the groundbreaking work by Iijima in 1991.^[^ ^32^ ^]^ CNTs consist of sp^2^ hybridized carbon atoms and are formed by the rolling up of graphene sheets into a seamless cylindrical structure. The hollow cylindrical structure of CNTs can be either open or closed by hemi‐fullerene caps. Furthermore, CNTs can be single‐walled or multi‐walled. Single walled CNT (SWCNT) is formed by rolling up of a single layer graphene sheet. SWCNT typically features a diameter of ≈1 nm.^[^ ^33^ ^]^ The properties of SWCNTs exhibit a strong dependence on the orientation of graphene sheets upon rolling. Three types of CNT configurations exists based on the orientation of the graphene sheets, namely Armchor, Zigzag and Chiral, which are responsible for metallic, semi‐conducting and semi‐metallic properties of CNTs, respectively.^[^ ^34^, ^35^ ^]^ When multilayer graphene sheets are rolled up, they form multi‐walled CNTs (MWCNT), where the distance between concentric layers of graphene cylinders is ≈3.4 Å, as shown in Figure 2c. MWCNTs feature a typical diameter ranging from 10 to 50 nm and a length ranging from 1 to 20 µm.^[^ ^34^ ^]^

#### 2.1.5 Amorphous Carbon, Glassy Carbon, and Carbon Dots

Amorphous carbon, as Robertson defined, is “a highly disordered form of carbon”.^[^ ^36^ ^]^ It features both sp^2^ and sp^3^ hybridized carbon and is characterized by the ratio of sp^2^ and sp^3^ site concentration, which, in many cases, depends on the fabrication process. Figure 2d shows an example of transmission electron microscopy (TEM) image of amorphous carbon obtained from pyrolysis of a biopolymer, where short‐range order could be seen originating from the sp^2^ carbons. Typically an evaporated or sputtered amorphous carbon film features 85% or more sp^3^ carbon bonds.^[^ ^37^, ^38^ ^]^ The carbon films featuring a very high (almost exclusively) concentration of sp^3^ carbon are called diamond‐like carbon (DLC) due to its high hardness originating from the sp^3^ hybridized carbons similar to diamond. The DLC films are typically hydrogenated, for better stabilization of the films to feature up to 50% of hydrogen in the material.^[^ ^37^ ^]^ The non‐hydrogenated DLC films are designated as tetrahedral amorphous carbon (taC).

Glassy carbon, often termed as vitreous carbon or glass‐like carbon (named by the International Union of Pure and Applied Chemistry ), is a nongraphitizable carbon, which is typically obtained through high‐temperature pyrolysis of an organic precursor.^[^ ^39^, ^40^ ^]^ It features sp^2^ hybridized carbon atoms, which form long‐range graphene sheets. However, unlike graphite, these graphene layers are randomly oriented, forming a turbostratic (misaligned basal planes) arrangement, as shown in Figure 2e. Along with the turbostratic graphene planes, it also features a significant amount of fullerene or fullerene‐like curved graphene layers.^[^ ^41^ ^]^ The turbostratic graphene layers and fullerene‐like structures form numerous voids and closed pores within the microstructure, which makes glassy carbon lightweight but impermeable to gases.

Carbon dots, also known as carbon quantum dots (CQDs), are quasi‐0D carbon nanoparticles, with an average size below 20 nm.^[^ ^42^ ^]^ An example of the electron microscope image of CQDs is shown in Figure 2f.^[^ ^43^ ^]^ Graphene quantum dots are mainly composed of sp^2^ hybridized carbon atoms and possess multi‐layer graphene sheets (see the inset of Figure 2f), which are connected with chemical groups at their edges or within the interlayer graphene planes. CQDs exhibit excellent fluorescence properties, which comes from the quantum confinement of these CQDs.

### 2.2 Properties Related to Scaffolding Materials

**Figure 3:**
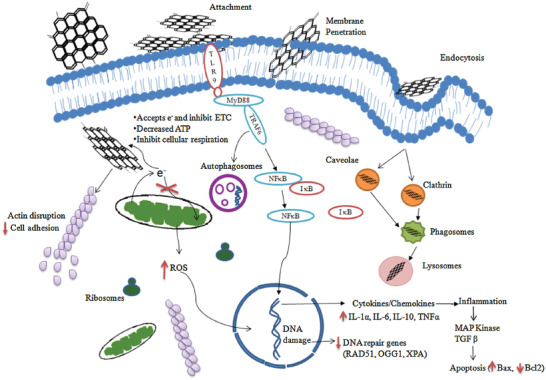
Mechanisms of cytotoxicity induced by graphene nanomaterials. Graphene interacts actively with the cell membranes and penetrates it due to its small size. Upon entry, it can result in an increase in the production of reactive oxygen species (ROS) by inducing oxidative stress. It can further inhibit electron transport chain, causing a depletion in cellular adenosine triphosphate (ATP) level. These phenomena can result in DNA damage and inflammation, which can further trigger cell apoptosis. Reproduced with permission.^[^ ^44^ ^]^ Copyright 2016, Elsevier.

The most important requirement of a scaffold material is that it should be biocompatible without suffering any inhibition by the immune system. All of the carbon allotropes exhibit bioactive properties without any need for surface functionalization. Toward specifically articular tissue engineering, the majority of carbon materials exhibit no or minimal cytotoxicity toward osteoblast cells. However, the size, shape, concentration, surface functionalization, and rate of aggregation of the carbon nano‐materials substantially impact the cytocompatibility of the carbon materials. Particularly for CNTs and graphene, several researchers have addressed the cytotoxic activities when used as scaffold materials.^[^ ^44^, ^45^ ^]^ It has been demonstrated that these nanomaterials can penetrate cell membranes, causing serious damage to the cell membranes. Further translocation into cells and cell nuclei can induce inflammation and even genotoxicity.^[^ ^46^ ^]^ **Figure** **3** illustrates different mechanisms of cytotoxicity induced by graphene nanomaterials.^[^ ^44^ ^]^ However, the majority of these cytotoxic responses was observed when CNTs or graphene were used as the nanomaterials and these nanomaterials could freely interact with cells. When these materials are used in composites and their free movement is restricted, the cytotoxic effects are found minimal or even none.^[^ ^46^, ^47^, ^48^ ^]^ Another method to attenuate the cytotoxicity is appropriate surface coating or functionalization. For instance, surface functionalization of graphene nanomaterials with polyethylene glycol or bovine serum albumin significantly reduced the toxicity to macrophages.^[^ ^49^ ^]^

Biomechanically speaking, carbon‐based materials cover the whole spectrum of interest for interacting with the human body, as already introduced and schematically shown in Figure 2. In tissue engineering, developing synthetic scaffolds, whose mechanical properties are fine‐tuned to those from the tissues being repaired, is fundamental: cells can feel the stiffness of their microenvironment and proliferate, grow, migrate and differentiate accordingly, as several studies have demonstrated.^[^ ^50^, ^51^, ^52^ ^]^ Typically, soft substrates mimicking biomechnical properties of brain are neurogenic; intermediate stiff substrates mimicking muscles are myogenic; and stiff substrates mimicking collagenous bone can induce osteogenic differentiation from mesenchymal stem cells.^[^ ^48^, ^53^ ^]^ Carbon allotropes cover a large range of tensile strength and Young's modulus values, and depend on the morphology of the carbon materials. For example, graphene nanomaterials enhance stem cell differentiation into osteoblastic lineage due to their high stiffness.^[^ ^48^, ^54^ ^]^ The high tensile strength (⩾50 GPa), high Young's modulus (⩾1 TPa), and high aspect ratio long tubular shape of CNTs make them highly suitable for collagen fibers.^[^ ^55^, ^56^, ^57^ ^]^ Apart from articular tissue regeneration, the bio‐mechanical versatility of carbon‐based materials has expanded other territories as well, including neural,^[^ ^58^ ^]^ skin,^[^ ^59^ ^]^ cardiac,^[^ ^60^ ^]^ musculoskeletal,^[^ ^61^, ^62^ ^]^ and chondral^[^ ^63^ ^]^ tissue engineering.

Carbon materials can be easily functionalized with different surface functional groups, which not only facilitates cell fixation on the carbon surface, but also allows to function in biodevices and drug delivery systems. For example, graphene can adsorb osteogenic inducers dexamethasone and β‐glycerolphosphate through π–π stacking, which can accelerate the osteogenic differentiation of mesenchymal stem cells.^[^ ^48^ ^]^ Surface functionalization further allows carbon nanomaterials to be used as reinforcing material in several ceramic and polymer‐based composite scaffold materials. For example, graphene can be transformed to GO through oxidation, which exhibits excellent hydrophilicity due to its surface functional groups. The presence of these functional groups also allows to disperse GO in several organic solvents and polymer matrices, facilitating the fabrication of high strength and stable composite scaffold structures.^[^ ^8^ ^]^ Good electrical conductivity of the carbon materials (except for diamond, which is electrically insulating) is an added advantage in tissue engineering applications. It can facilitate electrical stimulation to the culturing cells, which further allows for improved cell proliferation and osteogenic activity.^[^ ^64^, ^65^ ^]^ Carbon scaffolds may further exhibit self‐sensing activities during cell culturing due to the excellent electrochemical properties of the carbon materials. However, the self‐sensing property requires further investigation. Furthermore, carbon‐based materials exhibit excellent photothermal activity under light irradiation by resulting in a local increase in temperature. The photothermal properties of carbon materials allow to achieve the antimicrobial effect by hyperthermic killing of bacteria, which has been reviewed recently.^[^ ^66^, ^67^ ^]^ The synergic effect of photothermal and photodynamic activities of carbon materials can further facilitate wound healing, muscle repair, and noninvasive cancer therapy.^[^ ^67^, ^68^ ^]^

Besides, in the specific area of articular tissue engineering, the varied structures of carbon‐based materials can be engineered to achieve truly biomimetic spatial configurations and surface morphologies, including fibers–both compact and hollow–for ligaments, tendons, and muscular tubules;^[^ ^69^, ^70^ ^]^ spongy and interwoven meshes for chondral and subchondral defects;^[^ ^71^, ^72^ ^]^ and plates and 3D lattices and foams for cortical and trabecular bone.^[^ ^73^, ^74^ ^]^ The epigenetic cues provided by the morphology and topography of the cellular microenvironment synergize with those derived from the stiffness of the extracellular matrix and may reinforce the global regeneration strategy.^[^ ^75^, ^76^ ^]^

## 3 Carbon‐Based Materials in Tissue Engineering: State‐of‐the‐Art

We now review the application of different carbon‐based materials to the repair of varied articular tissues, providing a description of the current state‐of‐the‐art, which are summarized in **Table** **1**. Subsequent sections deal with more recent design strategies and combinations of carbon geometries, carbon materials and related manufacturing processes, aimed at solving the more complex challenges that appear when dealing with articular reconstructions involving several types of cells and tissues.

### 3.1 Carbon Fibers and Meshes

Early studies focusing on carbon materials as candidates for the reconstruction of damaged tissues explored the benefits and drawbacks of conventional carbon fibers as the most common industrially available carbon material together with graphite. The remarkable mechanical performance of carbon fibers and the feasibility of weaving them to obtain chords, meshes, and 3D constructs inspired strategies for application to ligament and tendon,^[^ ^77^, ^78^ ^]^ cartilage,^[^ ^71^, ^72^ ^]^ and bone repair.^[^ ^73^, ^79^, ^80^, ^81^ ^]^ From a surgical perspective, the textile‐like structure of these carbon fibers supported straightforward implantation procedures for surgeons. However, since the pioneering studies, biocompatibility was found to be adequate but not excellent, which leads to the incorporation of polymeric coatings,^[^ ^78^ ^]^ a common practice in medical implants like drug‐eluting polymer‐coated metallic stents, to mention an example.

Although initial stability after implantation was found remarkable, long‐term in vivo performance did not keep up with the high expectations, affected by aspects including biomechanical mismatches in relation to the tissues being repaired, brittleness, eventual fragmentation, or increased osteoarthrosis.^[^ ^10^, ^77^ ^]^ More recently, to progress toward improved long‐term performance, not only supporting polymeric coatings as improved biointerfaces for minimizing mechanical mismatches, but also carbon fiber‐polymer composites structures have been proposed,^[^ ^79^ ^]^ as further analyzed in Sections 4.1 and 5.1.

In any case, current trends in carbon‐based materials are exploring more modern options, within this large family, with very promising results, as described in the following subsections.

### 3.2 Carbon Nanotubes

**Figure 4:**
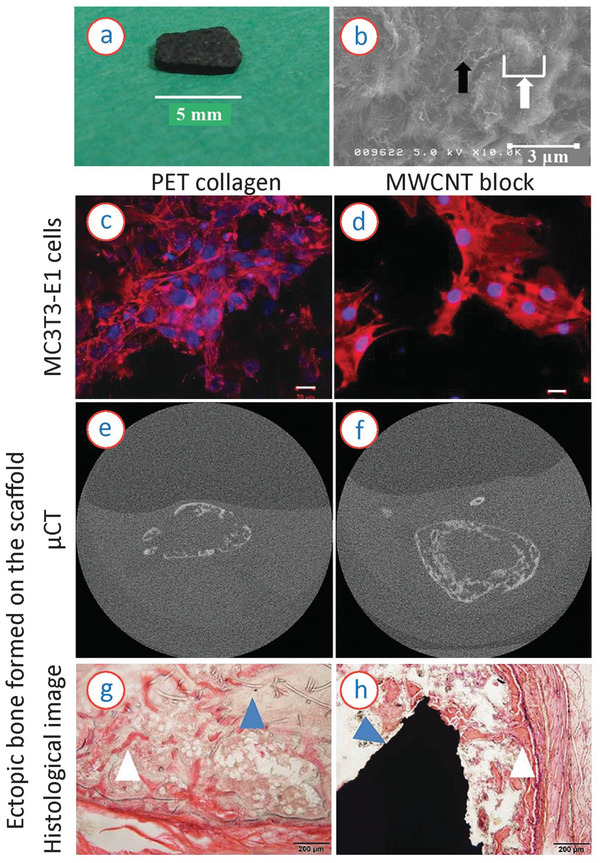
a) Photograph and b) scanning electron microscopy (SEM) image of a MWCNT block. The black arrow and white arrow in the SEM image represent nanosized irregularity formed by MWCNT fibers and microsized irregularity formed by an aggregate of MWCNT fibers, respectively. c–h) Comparison of the MWCNT block scaffold with a PET‐reinforced collagen scaffold. c,e,g) Results on the PET‐reinforced collagen scaffold and (d,f,h) on the MWCNT scaffolds. c,d) Cell culturing results on the scaffolds at 3 weeks after subcutaneous implantation of rhBMP‐2 loaded scaffolds in mice; e,f) μCT images of ectopic bone formed on the scaffolds; and g,h) histopathological image of subcutaneous ectopic bone formation in mouse. The white and blue arrow in (g) represent remnant PET fibers and newly formed bone, respectively. The white and blue arrow in (h) represent an MWCNT block and newly formed bone, respectively. The MWCNT showed better areal coverage of cells, yielding to more rigid and complete bone formation, without any debris within the newly formed bone. Reproduced with permission.^[^ ^82^ ^]^ Copyright 2017, PLOS.

CNTs and carbon nanocomposites, in which CNTs play a fundamental role, have emerged in the last years as an exciting alternative for carbon fibers and meshes in the tissue engineering arena. Their superior mechanical properties, lightweight structure, and tunable electromechanical behavior, among other aspects, make CNTs a suitable material in several research studies. Groundbreaking studies have already demonstrated the potentials of both SWCNTs and MWCNTs and their composites for the engineering of cartilage and bone (**Figure** **4**)^[^ ^56^, ^82^, ^83^, ^84^ ^]^ and of tendons and ligaments.^[^ ^56^, ^57^ ^]^ Their biomechanical similarity to collagen fibers has been highlighted, and their 3D processability through varied techniques, such as electrospinning, solvent casting, freeze‐drying, phase separation, and several rapid prototyping tools like bioprinting, digital light processing, laser stereolithography, or fused deposition modeling,^[^ ^83^ ^]^ expand their horizons for the personalized repair of multiple tissue types. Furthermore, they can be easily functionalized^[^ ^85^ ^]^ and hybridized with conventional carbon fibers and meshes to enhance their properties,^[^ ^86^ ^]^ which may also increase their versatility as biomedical materials.

Nevertheless, as happens with all nanomaterials employed for biomedical applications, their eventual harmful effects must be extremely carefully addressed in vitro before considering any in vivo actions. To cite an example, the European Medical Device Regulation (MDR 2017/745), in the classification rules of its Annex VIII, considers the incorporation of nanomaterials to the structure of medical devices as potentially harmful, and its Rule 19 states that, “all devices incorporating or consisting of nanomaterial are classified as class III if they present a high or medium potential for internal exposure; class IIb if they present a low potential for internal exposure; and class IIa if they present a negligible potential for internal exposure.^[^ ^87^ ^]^ From an ethical point of view, nanomaterials in general are studied under the scope of nanoethics, and interesting research has specifically dealt with the use of carbon nanotubes for biomedical applications.^[^ ^88^ ^]^ Apart from their unique properties, CNTs can cross the cytoplasmatic and nuclear membranes, which can be wisely applied to the development of innovative therapies, beyond the tissue engineering field, including delivery of anticancer therapies, genetic treatments, or DNA to the nucleus.^[^ ^89^ ^]^ However, concerns about their toxicity associated with cellular membrane disruption, and possible carcinogenic effects in the respiratory system have also been reported.^[^ ^90^, ^91^ ^]^ Particularly for articular tissue engineering, the cytotoxic effects of CNTs have been reported to have an negative impact on the viability of primary osteoblast cells and inhibit the mineralization of osteoblasts in a time‐ and dose‐dependent manner.^[^ ^92^, ^93^ ^]^ The order of cytotoxicity was found to be SWCNTs > bi‐layer CNTs> MWCNTs. However, these cytotoxicity effects can be minimized or even eliminated by appropriate surface functionalization or using composites of CNTs as the scaffold materials.^[^ ^47^, ^94^ ^]^

These ethical, legal, and safety considerations apply to other carbon‐based materials and to all carbon nanocomposites, as further analyzed in Section 5.5 on safety and regulatory issues.

### 3.3 Graphene and Graphene‐based Materials

Graphene, GO, and rGO have attracted the attention of tissue engineering researchers for more than a decade: their electrical conductivity is relevant for neural tissue engineering studies; their 2D structures are biomimetic for skin regeneration; their surface functionalization capabilities using biomolecules are outstanding, which promotes their possibilities as innovative biointerfaces; and their astonishing mechanical properties can be used to synthesize graphene‐based composites with remarkable mechanical strength,^[^ ^96^ ^]^ as further discussed in Section 4.1 that deals with polymer–carbon and ceramic–carbon nanocomposites. The electromechanical properties of these materials may also promote smart responses and self‐sensing solutions for tissue engineering, as analyzed in Section 6.3.

**Figure 5:**
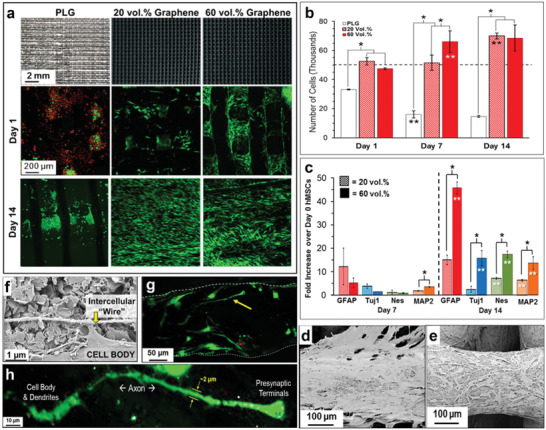
Example of osteogenic behavior of graphene scaffolds. a) Top row: Photographs of 3D printed scaffolds of PLG materials with 0%, 20%, and 60% graphene (volume %); Scanning laser confocal 3D reconstruction projection of live (green) and dead (red) human mesenchymal stem cells (hMSCs) cultured at day 1 (middle row) and day 14 (bottom row) on the scaffolds. b) Number of hMSCs present on the scaffolds at day 1, 7, and 14, suggesting superior cell proliferation on the scaffolds with higher concentration of graphene. c) Neurogenic relevant gene expression of the cells on the graphene scaffolds at day 7 and 14, showing better gene expression with higher content of graphene. d,e) SEM of hMSCs on the 20% and 60% graphene loaded scaffolds, respectively. f) High magnification SEM of hMSCs cell on 60% graphene scaffold at day 7, showing hMSCs connecting via a long “intercellular” wire. g) Scanning laser confocal 3D reconstruction of live (green) and dead (red) hMSCs cells on day 14 for 60% graphene scaffold. h) High magnification image of the cell indicated by yellow arrow in (f) showing the detailed features. Reproduced with permission.^[^ ^95^ ^]^ Copyright 2015, American Chemical Society.

In the specific area of articular tissue engineering, for the repair, reconstruction, and regeneration of bone, cartilage, tendons, and ligaments, graphene and graphene‐based materials are also being considered as candidate materials for challenging the status quo. Recent research efforts linked to graphene scaffolds for tissue engineering have been previously reviewed.^[^ ^97^ ^]^ The osteogenic behavior of graphene has been reported (**Figure** **5**),^[^ ^48^, ^95^, ^98^ ^]^ as well as challenges for its final application, including potential toxicity at higher concentrations and a nonbiodegradable nature.^[^ ^98^ ^]^ As regards the more challenging regeneration of cartilage (as compared with bone), graphene seems to provide stem cells with the right cues: researchers have demonstrated the usability of graphene oxide flakes as growth factor delivery carriers, to enhance chondrogenic differentiation of human mesenchymal stem cells in 3D hydrogels.^[^ ^99^ ^]^ In another example, nanocomposites of rGO and hydroxyapatite (HAp) promoted the osteogenic differentiation of pre‐osteoblasts and mineralization of calcium and phosphate without any inhibition to their proliferation, stimulating new bone formations.^[^ ^100^ ^]^ Graphene‐ and carbon nanotubes‐based materials also show promising potential for articular restorations, in which both bone and cartilage need repair,^[^ ^101^ ^]^ although similar concerns to those reported for nanotubes, and already mentioned for graphene, arise, particularly regarding long‐term performance and biological response. These graphene‐based nanomaterials are also being explored as reinforcements in polymer–carbon nanocomposites for ligament and tendon repair.^[^ ^102^ ^]^

Toward achieving truly 3D graphene‐based scaffolds, apart from the possibility of resorting to graphene‐based nanocomposites, the possibility of synthesizing graphene foams has been researched in connection with cardiac^[^ ^103^ ^]^ and neural^[^ ^104^ ^]^ tissue engineering applications. In both areas, enhanced cell proliferation and superior gene expression for the desired applications have been reported for the 3D graphene foams, when compared to conventional 2D graphene. These foams have been synthesized by a chemical vapor deposition process using Ni templates and initially described in previous studies.^[^ ^105^ ^]^ Alternative routes toward 3D and even 4D graphene are discussed in Sections 4.4 and 6.2. In any case, the experience acquired in 3D graphene foams for cardiac and neural tissue engineering could be arguably transferred to engineering innovative bone, cartilage, tendon, and ligament substitutes to contribute to complex articular restorations, probably synergizing with other carbon materials families, as discussed along Section 4.

### 3.4 Carbon Dots

The near 0D nature of carbon dots (or C‐dots), with characteristic sizes of less than 10 nm,^[^ ^106^ ^]^ prevents their application as structural materials for repairing large defects in articular injures and for the tissue engineering field in general. However, their charming properties make them excellent companions to other structural materials for progressing toward highly innovative diagnostic procedures and therapies. Emerging applications in different fields, including medicine, energy, and ecology, derive from characteristic photo‐induced electron transfer, photoluminescence, promising biocompatibility (although with similar considerations as those previously mentioned for CNTs and graphene), and low cost.^[^ ^42^, ^106^, ^107^ ^]^ Their medical applications as drug delivery materials and in theranostics are most analyzed, although their biocompatibility and eventual toxic effects are still a matter of debate, with positive and negative results both in vitro and in vivo,^[^ ^108^ ^]^ which emphasizes the importance of performing further research before their large‐scale transfer to patients.

Tissue engineering scaffolds may also benefit from incorporating C‐dots, as some researchers have already highlighted. For instance, it has been recently demonstrated that C‐dots are capable of both tracking and enhancing the osteogenic differentiation of mesenchymal stem cells, which proves relevant for the future of bone tissue engineering.^[^ ^109^ ^]^ Used as functionalizing elements for other biomaterials, they prove adequate for supporting and tracking cellular activities.^[^ ^110^ ^]^

Their role as electroconductive nanobiomaterials for tissue engineering and regenerative medicine has also been put forward, with an apparent superior biocompatible response of C‐dots over metallic quantum dots,^[^ ^111^ ^]^ which may in the future lead to smart synthetic scaffolds, extracellular matrices, or cell niches, as further discussed in Section 6.2.

### 3.5 Glassy Carbon

Glassy or vitreous carbons can be biomechanically adjusted and three‐dimensionally processed to achieve biomimetic porous reticular structures with attractive features, especially for trabecular bone tissue engineering. For example, reticulated vitreous carbon, commercially available as Duocel RVC foam, has been tested both in vitro and in vivo, demonstrating excellent adhesion of mesenchymal stem cells and primary chondrocytes and bone regeneration properties, but incompatibility with cartilage regeneration,^[^ ^112^ ^]^ probably due to excessive stiffness. The low‐cost manufacturing of reticular glassy carbon using templates of sucrose has been studied with remarkable results in terms of interconnected porosity, bone‐like morphology, compressive strength, and cytocompatibility, assessed with human osteoblasts.^[^ ^113^ ^]^ Sol–gel processes for functionalizing glassy carbon lattices with bioglass have been also proposed and shown in vitro the potential presence of the initial phases of the apatite formation process, after 21 days under culture.^[^ ^114^ ^]^ The creation of hydroxyapatite scaffolds, using a multi‐step “from wood to bone” process, involving the pyrolysis of ligneous raw materials to produce vitreous carbon templates with natural and complex anisotropic pore structure, followed by carburization, carbonation, and phosphatization, constitutes another successful strategy for the production of bioinspired bone repair structures.^[^ ^115^ ^]^

**Figure 6:**
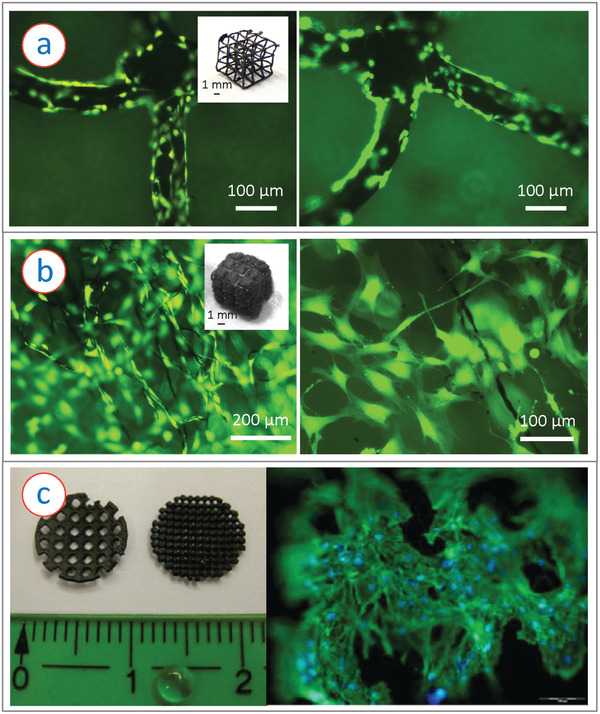
a) Osteoblast like murine MC3T3‐E1 cells cultured on additively manufactured glassy carbon microlattice structures on day 3 after cell seeding, showing good cytocompatibility of the cells. Inset shows example of a glassy carbon architecture. Adapted with permission.^[^ ^116^ ^]^ Copyright 2020, Wiley‐VCH GmbH. b) The murine MC3T3‐E1 cells cultured within a carbon fiber filled glassy carbon microlattice architecture (inset showing a photograph of the hybrid structure). The cells were proliferated not only over the microlattices and along the carbon fibers (left), the cells also established inter‐cellular connections within the voids between adjacent fibers (right), yielding a 3D cell colonization. Reproduced with permission.^[^ ^117^ ^]^ Copyright 2021, Elsevier. c) Left: Example of DLC coated rapid prototyped scaffolds. Right: Cell growth validation of the DLC coated scaffolds by culturing hMSCs cells on the scaffolds. Reproduced with permission.^[^ ^118^ ^]^ Copyright 2011, Wiley‐VCH GmbH.

Our own team has focused on the design‐controlled production of glassy carbon scaffolds for tissue engineering, by pyrolysis of complex‐shaped biomimetic stereolithographic templates (**Figure** **6**a),^[^ ^116^ ^]^ as further discussed in Section 4.2. The possibility of achieving 3D multi‐scale glassy carbon, with a structural lattice filled by softer microfibers, has been also demonstrated (Figure 6b).^[^ ^117^ ^]^ These functionally graded structures show potentials toward biomimetic designs, in which a structural lattice may help to regenerate bone, while fiber meshes and micrometric carbon threads may support the reconstruction of cartilage and fibrillar tissues, although further research is needed to demonstrate the repairability and longevity of large defects involving varied tissues.

### 3.6 Nanodiamonds and Diamond‐Like Carbon

The beauty and perfection of diamond is also starting to have an impact in tissue engineering and biotechnology in general, as previously reviewed.^[^ ^119^ ^]^ As the authors explain, for advanced biomedical applications, diamond is especially promising in nanostructured arrangements, basically as nanoparticles, nanostructured diamond films, and composite scaffolds with a matrix containing diamond nanoparticles as fillers, also known as nanodiamond‐loaded nanofibrous scaffolds. Their effects have been tested with bone‐derived cells and shown potentials for bioimaging, biosensing, and drug and gene delivery, as a complement to tissue engineering structures. However, the number of reports on nanodiamond cytotoxicity is increasing, with reviewed studies including in vitro and in vivo studies.^[^ ^119^ ^]^ Once again, it is necessary to highlight the need for systematically assessing in vitro any potential harmful effect of these innovative nanomaterials combinations, before proceeding to in vivo tests. Proceeding according to ISO standard 10993 on biocompatibility assessment^[^ ^120^ ^]^ is the internationally recognized option of choice, as additionally discussed in Section 5.5, which deals with safety and regulatory issues.

Regarding diamond‐like carbon (DLC) coatings, their benefits for improving the in vitro biological response of polymeric and metallic tissue engineering scaffolds and implants have been investigated (Figure 6c).^[^ ^118^, ^121^, ^122^ ^]^ These coatings tend to enhance cell adhesion and provide a remarkable surface hardness, thanks to their high content of sp^3^ hybridization, as well as an adequate corrosion resistance against chemicals, abrasion endurance, good biocompatibility, and uniform flat surface.^[^ ^123^ ^]^ However, their long‐term performance may be affected by adhesion or delamination problems, as happens with thin‐film technologies in general. Beneficial synergies can be obtained by combining highly precise additive manufacturing technologies, capable of creating complex‐shaped biomimetic objects, but sometimes employing photopolymers and resins with very limited biocompatibility, with the enhanced biological response provided by the DLC coatings for performing systematic in vitro studies.^[^ ^118^ ^]^

## 4 Fabrication Strategies of Carbon‐Based Materials for Articular Tissue Engineering

**Figure 7:**
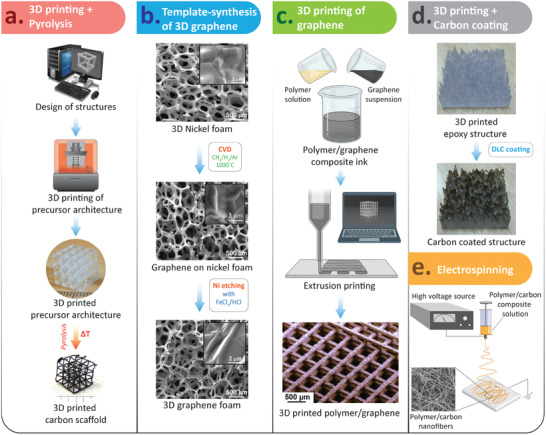
Illustrations of different approaches used for the fabrication of carbon‐based scaffolds: a) 3D printed scaffolds of glassy carbon using stereolithography and subsequent pyrolysis; b) 3D graphene foam synthesized using CVD on a nickel template, followed by nickel etching; c) 3D printing of graphene scaffolds; d) DLC coating over a 3D printed epoxy scaffold; and e) electrospinning of composite‐fibers for textile and fabric based scaffolds. (b) Adapted with permission.^[^ ^104^ ^]^ Copyright 2013, Springer Nature. The bottom sub‐figure in (c) is reproduced with permission^[^ ^95^ ^]^ Copyright 2015, American Chemical Society.

Although different carbon‐based materials have proven useful for the study and repair of damaged articular tissues, both in vitro and in vivo, the previously reviewed solutions (see Section 3) have normally employed a single type of carbon, in most cases with a homogeneous structure, for healing a specific tissue. Typically, fibers have been applied to tendons and ligaments, meshes and foams to cartilage and lattices, or plates to bone. However, to reach truly holistic solutions for articular repair, capable of engineering damaged regions, in which bone and cartilage, bone and tendon, or bone and ligament are involved, it is necessary to progress toward multi‐scale and functionally graded solutions benefiting from artificial constructs made of carbon‐carbon (micro‐/nano‐) composites. In addition, to increase the degree of versatility needed for complex and personalized articular reconstructions, these carbon–carbon composites, and carbon‐based materials in general, may benefit from eventually incorporating polymers and ceramics to some extent, as matrices or as reinforcing elements. The emergence of the carbon micro‐electromechanical systems (C‐MEMS) technology,^[^ ^40^, ^124^ ^]^ the discovery of fullerenes^[^ ^125^ ^]^ and carbon nanotubes,^[^ ^32^ ^]^ and the rise of graphene technology,^[^ ^18^ ^]^ among others, have provided a plethora of innovative methods for synthesizing and processing carbon materials for extremely varied application fields. This section describes their hybridization with other technologies (**Figure** **7**), derived from parallel advances in nanocomposite synthesis, and in additive manufacturing processes, to achieve multi‐scale and functionally graded carbon‐based scaffolds with interesting features and potentials for improved articular tissue engineering.

### 4.1 Polymer‐Carbon and Ceramic‐Carbon Nanocomposites

The relevance of biopolymers for the birth and early evolution of tissue engineering is outstanding, due to their interesting biocompatibility, with bioinert and biodegradable options; to the possibility of adjusting their properties, by including additives or modifying the polymerization conditions; and to their processability employing a wide set of technologies, from fused deposition modeling and extrusion‐based processes, to injection molding and hot embossing, to cite a few. However, in many cases, their mechanical performance lacks the necessary strength for weight bearing joints. Hence, the use of polymer matrix composites as scaffolding materials is a common option in modern tissue engineering.

Recently, combinations of carbon materials and polymers have been explored, leading to innovative polymer–carbon micro/nanocomposites for tissue engineering. Apart from the mechanical benefits of carbon materials for reinforcing polymers, their addition to the polymeric matrix can lead to conductive properties for enhanced transmission of electrical signals between the cells. Electrically conductive chitosan/carbon scaffolds^[^ ^126^ ^]^ and carbon‐based nanotube–gelatin scaffolds^[^ ^127^ ^]^ for cardiac tissue engineering have been proposed. Similar combinations may be used for load bearing constructs with self‐monitoring capabilities. Remarkable results in terms of electrically conductive scaffolds have also been achieved by combining hydrogels with microengineered hollow graphene tube systems, which may apply to biohybrid robotics.^[^ ^128^ ^]^ Their potentials for articular tissue engineering, especially for developing smart cartilage, should also be explored, due to the possibility of encapsulating stem cells within hydrogels, which can be also loaded with growth factors to promote cell differentiation into a chondrogenic phenotype.^[^ ^129^ ^]^ Several fabrication routes have been explored to obtain such composite materials, among which popular methods include 3D printing and electrospinning. 3D printing is majorly used for design‐controlled fabrication of the scaffolds, which is discussed in Section 4.4. Electrospinning, which is a technique to draw nanofibers from a polymeric droplet under a high electric field, is majorly used to obtain nanocomposite fibers and mats (Figure 7e), which is discussed in Section 3.1.

Regarding bone repair, ceramics provide more biomechanical scaffolds than polymers, and they can also benefit from being combined with carbon materials. For example, microfiber composites of self‐entangled CNTs, and bioactive nanoparticles (hydroxyapatite and bioactive glass), have been found highly suitable for growing cells such as osteoblasts and fibroblasts.^[^ ^13^ ^]^ The electrophoretic deposition of hydroxyapatite upon ultralight aerographite has also been demonstrated and their benefits for promoting osteoblastic activity has been assessed.^[^ ^130^ ^]^ These pioneering studies on carbon‐ceramic micro/nanocomposites for tissue engineering put forward the relevance of systematically combining materials and manufacturing/processing technologies for progressively approaching the complexity of the cellular microenvironment and better mimicking human nature.

### 4.2 Pyrolysis of Naturally Occurring and 3D Printed Cellular Precursors

Pyrolysis refers to high temperature heat treatment under the absence of oxygen. It is one of the most popular methods to transform a polymeric precursor to a carbonaceous material. During pyrolysis, the polymeric precursor undergoes a thermochemical decomposition, in which a majority of the non‐carbon elements present in the polymeric chain escapes the precursor matrix in the form of volatile byproducts, leaving behind a carbon‐rich material. The thermochemical cleaving of the polymeric chain typically occurs in the temperature range 300 to 450 °C. At this point, an amorphous carbon material is obtained. Upon further heating to a higher temperature, the carbon atoms in the matrix start rearranging themselves to yield a more ordered microstructure, yielding a glassy carbon material.^[^ ^41^ ^]^ The most important characteristics of the pyrolysis process is that the resulting carbon material retains the overall geometry of the precursor material, even though a significant structural shrinkage occurs during the pyrolysis process due to the release of the gaseous byproducts.^[^ ^131^, ^132^ ^]^ Such a characteristic not only allows to choose a naturally occurring precursor with a microstructure suitable for tissue engineering application, but also encourages to design customizable structures for a personalized tissue regeneration framework. For example, Tampieri et al. introduced the “from wood to bone” concept, where they pyrolyzed natural woods (Pine and rattan) to restore their hierarchically porous microstructure, which mimicked bone structures.^[^ ^115^ ^]^ This hierarchically porous carbon template was further transformed into a biomimetic and hierarchically organized hydroxyapatite scaffold by subsequent calcium carburization, oxidation, carbonation, and phosphatization. As mentioned in Section 3.1, carbon fibers and meshes are among the promising candidates for tissue regeneration in cartilage and bone repair. The most traditional technique used for the fabrication of carbon fibers is electrospinning followed by pyrolysis. In electrospinning, nanofibril mats are fabricated by drawing nanofibers from an polymeric solution under a high electric field. The fabricated nanofibers are converted to carbon nanofibers through the pyrolysis steps. The majority of the studies where carbon fibers, by themselves or in a composite form, were used as the scaffold material for tissue engineering, used the electrospinning‐pyrolysis route for production of the carbon nanofibers.^[^ ^80^, ^81^, ^133^ ^]^ Pyrolysis of bacterial cellulose was also employed to achieve carbon nanofibers toward bone tissue regeneration.^[^ ^134^ ^]^

Our team has demonstrated the integration of 3D printing technology with a subsequent pyrolysis process toward design‐controlled fabrication of glassy carbon scaffolds for tissue engineering applications (Figure 7a).^[^ ^116^ ^]^ 3D printing facilitates the design and fabrication of complex 3D geometries, which are challenging in other more traditional fabrication processes. In our approach, we first fabricated 3D complex microlattice architectures of an epoxy resin using a stereolithographic 3D printing technique. Upon pyrolysis, these 3D structures were not only converted into 3D microlattice architectures of glassy carbon, but the associated shrinkage process resulted in an geometrical resolution significantly higher than the original stereolithographic process. Even though these carbon architectures exhibited excellent cytocompatibility, the spacing among the microlattices was too large to establish intercellular connectivity. To overcome this, we demonstrated the filling of precursor architectures with softer cotton microfibers. Pyrolysis of the hybrid structures led to the formation of multi‐scale functionally‐graded 3D carbon architectures.^[^ ^117^ ^]^ The 3D printed glassy carbon served as a rigid framework, and the cotton‐derived flexible carbon fiber entanglements introduced compressibility to the structure, as well as creating microenvironments for cell interactions. Such hybrid structures exhibited higher degrees of cell growth. As mentioned earlier, the pyrolysis‐assisted fabrication of functionally graded structures are promising toward biomimetic designs, in which a structural lattice may help to regenerate bone, while fiber meshes and micro‐metric carbon threads may support the reconstruction of cartilage and fibril tissues.

### 4.3 Template‐Assisted Fabrication

Apart from pyrolysis, template‐based fabrication processes have emerged as alternative routes to achieve 3D construction for tissue regeneration. Typically, it involves the growth of thin films of carbon material on a 3D template, followed by etching of the template material. For example, 3D graphene foams (3DGF) were synthesized via chemical vapor deposition (CVD) growth on a 3D nickel foam, followed by etching of the nickel template (Figure 7b). Such 3DGFs exhibited spontaneous neuronal and osteogenic differentiation of mesenchymal stem cells.^[^ ^104^, ^135^ ^]^ Selhuber‐Unkel and her team used zinc oxide (ZnO) tetrapod structures as the template, upon which carbon or CNT layers were deposited using CVD or a dip‐coating technique. The ZnO was further etched to produced aerographite or CNT tube (CNTT) structures.^[^ ^13^, ^130^, ^136^ ^]^ An advantage of the template‐assisted fabrication route is that it allows to retain the 3D microenvironment created by the template even after etching it away. The method does not suffer from the shrinkage phenomenon associated with pyrolysis, which yields collapse of the 3D microstructure beyond a critical dimensional limit. The 3D microenvironments of the carbon structures obtained in template‐assisted routes advantageously impacts signal transduction, intercellular interactions, and tissue regeneration.^[^ ^137^ ^]^ On the other hand, these template‐assisted routes suffer from high production costs due to more complex and expensive equipment and infrastructure, and the need for highly trained personnel, which limit the methods only to laboratory scale trials.

### 4.4 3D Printing of Graphene and CNT

The mergence of 3D printing technologies has led to a particular focus on the design‐controlled fabrication of customizable scaffolds for tissue engineering, particularly for bone and articular tissue repair.^[^ ^138^ ^]^ In the previous section, we have briefly described our recent progress on the fabrication of 3D printed glassy carbon scaffolds. In Sections 3.2 and 3.3, we have also described how CNT and graphene‐based materials have exhibited interesting and promising results for bone and articular tissue engineering. However, it should be noted that CNT, graphene, and graphene oxide cannot be directly 3D printed using any of the current the state‐of‐the‐art additive manufacturing technologies. Therefore the most traditional method to process these materials using 3D printing, is to disperse them into a synthetic scaffolding material to prepare a composite feedstock suitable for 3D printing (Figure 7c). The scaffolding materials used to host the CNT, graphene, or GO for bone and articular tissue regeneration include metals (e.g., titanium), bioceramics (e.g., calcium phosphates, hydroxyapatite, and bioactive glass), and natural or synthetic polymers (e.g., poly‐lactic acid (PLA), polyvinyl alcohol (PVA), poly‐glycolic acid (PLGA), polycaprolactone (PCL), poly(propylene fumarate) (PPF), alginate, chitosan, gelatin, and collagen).^[^ ^139^, ^140^ ^]^ However, to date no bio‐ink that targets live cells within the ink matrix is available that simultaneously contains these carbon materials. Upon fabrication of the composite scaffold, cells are seeded on to the scaffold in an appropriate growth medium for further proliferation or differentiation.

The properties of the 3D printed scaffolds not only depend on the design features of the scaffold, but also on the 3D printing process. The most popular 3D printing methods for fabrication of CNT, graphene, or GO based scaffolds include fused deposition modeling (FDM), selective laser sintering (SLS), and syringe based extrusion methods. SLS is mainly used for ceramic and metal based scaffolds, whereas FDM and syringe‐based deposition are mainly used for the polymer based composites.^[^ ^139^, ^140^ ^]^ Laser stereolithography (STL) methods are also emerging as a high resolution and rapid technique for polymer‐based scaffold fabrication.^[^ ^101^, ^141^ ^]^ SLS, FDM, and STL methods allow better controllability of the design, influencing the mechanical properties of the scaffold. However, they are not compatible with bio‐inks due to the high temperature involved in FDM extrusion, or the high energy involved in SLS or STL processes for sintering and cross‐linking. On the other hand, syringe based methods are promising for bio‐inks, even though they suffer from low resolution. The extrusion based 3D printing methods (FDM and syringe‐based) further exhibit better mechanical and electrical properties, due to the self‐aligning phenomenon of graphene sheets and CNTs during extrusion.^[^ ^142^ ^]^

### 4.5 Coating of Carbon Nanomaterials

Tissue engineering advances, not only focusing on implantable constructs for repair or regeneration, but also on performing in vitro studies with cells cultured upon scaffolding structures and microfluidic devices, like labs‐ and organs‐on‐chips, are able to recapitulate the complexity of many different physiological structures and to model the origins and genesis of a wide set of diseases. The remarkable biocompatibility of several carbon materials makes them adequate for supporting cell adhesion and growth in cell culture systems. In some cases, the complexity and precision required for scaffolding structures leads to selecting high‐performance freeform fabrication techniques (i.e., laser stereolithography, digital light projection, two‐photon polymerization), which work with materials like epoxy‐based and methacrylate‐based photopolymers, whose biocompatibility is seldom adequate for working with cells. These scaffolds, if adequately coated with thin films of carbon, can constitute interesting cell niches for in vitro studies. For example, the combination of laser stereolithography with DLC has proven useful for studying the impact of topography on cell behavior (Figure 7d).^[^ ^118^ ^]^ DLC has also improved the biocompatibility of segmented polyurethane scaffold sheets,^[^ ^121^ ^]^ and its benefit for improving the biological response of conventional implants has been also praised.^[^ ^143^ ^]^ More recently, Ti‐doped DLC films have shown improvements in terms of elastic modulus, hardness, adhesion strength, and surface roughness of the coating, applied upon Ti substrates, while maintaining its biocompatibility.^[^ ^144^ ^]^ Authors envision future studies dealing with exploring additional combinations of additively manufactured scaffolds, for instance obtained using powder‐based laser fusion of Ti alloys, with DLC coatings for enhanced biocompatibility.

Diamond‐like carbon coatings are well established in the biomedical industry, but current research trends are also dealing with the application of carbon nanotube coatings. For instance, bioglass‐based scaffolds with carbon nanotube coating, obtained by electrophoretic deposition, have been proposed for bone tissue engineering^[^ ^145^ ^]^ and dense single‐walled carbon nanotube coatings have promoted osteogenic differentiation of mesenchymal stem cells, which generates interest for articular and dental substitutes.^[^ ^146^ ^]^

### 4.6 Textile‐Based Techniques

Several studies have demonstrated that knitting fibers is an excellent procedure for achieving 3D tissue engineering scaffolds with remarkable mechanical behavior and adequate microstructure, useful for repairing tendons, ligaments, and cartilage.^[^ ^147^ ^]^ PLLA and PLGA, polymeric materials common in 3D printing by fused deposition modeling, have been processed as fibers and knitted for varied tissue engineering applications,^[^ ^147^ ^]^ and other biotextiles such as PBS and silk have also been studied.^[^ ^148^ ^]^ Considering that the knitting of carbon fibers is a mature technology, it may be transferred almost directly to the manufacture of scaffolding superstructures, as previously proposed.^[^ ^149^ ^]^ Pyrolyzed cotton or carbon cotton has been investigated of late as a material for providing a soft microenvironment for cell culture applications.^[^ ^117^ ^]^ Further investigations of pyrolysis of knitted cotton, for achieving design‐controlled patterns capable of guiding cell growth and tissue differentiation, may also contribute to the portfolio of materials for articular tissue engineering.

Combining conventional 3D printed frameworks, like those achieved by fused‐deposition modeling of PLA, with knitted carbon fibers, is another example of how textile‐based techniques apply to the tissue engineering field. The filling or knitting of carbon fibers can increase the mechanical strength of the original construct, while helping to provide the cells with a truly 3D microenvironment for enhanced scaffolding colonization. The alignment of the fibers helps cells to adopt fibrillar configurations, which is promising for tendon and ligament repair.^[^ ^150^ ^]^

## 5 Current Challenges toward Clinical Application

The growing family of carbon‐based materials and carbon nanocomposites is already catching the attention of researchers in the tissue engineering field, with several studies showing promising results for articular repair and regeneration, as reviewed in previous sections. However, as happens still with most scaffold‐based solutions for regenerative medicine, reaching patients and promoting sustainability is still challenging and uncommon.^[^ ^151^ ^]^ The usual requirement of biodegradability for scaffolding materials, especially in the early years of the field, may have biased the selection of biomaterials toward very specific realms (i.e., bioabsorbable polymers) and diverted the attention from the key requirements. Among these key requirements, recapitulating the architecture of the cellular microenvironment, and matching the mechanical performance of the original tissue and biocompatibility, are fundamental, while biodegradability plays a secondary optional role.^[^ ^151^ ^]^ Several types of carbon‐based materials fulfill the fundamental requirements for becoming the solution of choice for repairing and regenerating bone, cartilage, tendon, and ligament. However, some challenges must still be tackled to achieve successful and sustainable clinical application, as detailed in the following subsections.

### 5.1 Long‐Term In Vivo Performance of Carbon‐Based Solutions

Biocompatibility is essential for tissue engineering scaffolds and biomaterials. Some previously referenced studies have put forward a suboptimal long‐term biocompatibility of carbon fibers for ligament or tendon repair, and discussed potential health risks of carbon nanotubes.^[^ ^10^, ^77^, ^90^, ^91^ ^]^ The promising biocompatibility of other families, like glassy carbons, graphene, and carbon nanocomposites, still needs to be further characterized and assured before clinical application can be attempted. In general, an adequate route for these evaluations is applying the ISO 10993, by testing cytotoxicity, sensitization, irritation, intracutaneous reactivity, subchronic toxicity, genotoxicity, implantation, and hemocompatibility. Nevertheless, even if the biomaterial or scaffold passes the criteria ISO 10993, long‐term performance may be affected by varied physicochemical phenomena within the organism. In the case of biodegradable implants, including biodegradable scaffolds, the rationale is to design or synthesize a material whose biodegradability is fine‐tuned to the healing process, providing mechanical support to the injured regions, and degrading away during or after healing.^[^ ^152^ ^]^ However, in the case of non‐biodegradable implants or scaffolds, corrosion, and degradation may lead to accumulating biomechanical failure and even to toxicity problems over years, as previously reported for metallic implantable devices.^[^ ^153^ ^]^ Within tissue engineering, for example, the mechanical performance and biological responses of 3D printed polymeric, ceramic, and metallic scaffolds during degradation have been systematically studied.^[^ ^154^, ^155^ ^]^ Regarding carbon‐based materials for hard and soft tissue repair, the most comprehensive biological evaluations have been performed with fabrics for which biological behavior dependent on the type of fibers employed was found to apply, that is, some of which may not be easily resorbable.^[^ ^156^ ^]^ Eventual fragmentation of carbon fibers used for tissue engineering has also been considered as a source of inflammation and osteoarthritis.^[^ ^10^, ^77^ ^]^ Even though carbon‐based materials are hard and stiff in nature, recent studies have shown the possibilities of biodegradation of carbon‐based materials, particularly graphene and CNTs.^[^ ^46^, ^157^ ^]^ Degradation of graphene and CNTs can be induced by strong oxidative enzymes or hydrogen peroxide (H_2_O_2_) treatment. H_2_O_2_ generally attacks the oxygenated defect sites, causing fragmentation of the carbon nanomaterials. This degradation mechanism has shown complete dissolution of 3D graphene scaffold in about 1 year.^[^ ^158^ ^]^ Pristine CNTs are more resistant to degradation due to their low defect sites. The surface functionalization of CNTs greatly influences the peroxide‐induced degradation process.^[^ ^157^ ^]^ However, MWCNTs exhibits higher resistance than SWCNTs due to their concentric graphitic structure. The degradation rate can be tuned by the types of surface functions and the concentration of defect sites. Furthermore, initial studies showed that the byproducts (mostly fragments of carbon nanomaterials) originating from the degradation process did not show any inflammatory responses.^[^ ^159^ ^]^ Although these studies show promising results, further systematic studies are needed before clinical uses of these materials. Furthermore, no biodegradability study is found for glassy carbon, DLC, or fullerene, which opens up opportunities for researchers to investigate the biodegradability of these carbon allotropes.

Another aspect affecting long‐term viability of scaffolding materials is their fatigue performance, which is intrinsic to their biocompatibility: a mechanically inadequate biomaterial or medical device cannot be considered biocompatible. Early studies, dealing with glassy carbon structures as implants, focused on comparing their performance with that of ceramic materials and studied their fatigue behavior, in parallel with their biocompatibility, with promising results.^[^ ^160^ ^]^ More recently, the impressive fatigue behavior of graphene, graphene oxide and graphene‐embedded nanocomposites has been studied.^[^ ^161^ ^]^

The remarkable superelasticity and high fatigue resistance of carbon aerogel materials^[^ ^162^ ^]^ may promote them as an interesting option for cartilage repair. In fact, carbon aerogel coatings have been successfully applied as coatings for enhancing beta‐tricalcium phosphate structures used for osteosarcoma therapy and bone regeneration,^[^ ^163^ ^]^ which validates their suitability for articular tissue regeneration.

### 5.2 Microvascular Complexity in Carbon‐Based Solutions

A major unsolved challenge in tissue engineering is the artificial creation of a truly functional and biomimetic vasculature enabling the transport of nutrients and the elimination of debris within 3D constructs.^[^ ^164^, ^165^ ^]^ Different strategies with encouraging findings coexist, from the combined use of cells, growth factors, cytokines, peptides, and proteins to generate new vessels, to scaffold‐based techniques relying on the combination of multi‐scale design, simulation and fabrication processes for recreating microvascular complexity.^[^ ^164^, ^165^ ^]^ In articular repair, cartilage is an avascular tissue (one of the reasons why its regeneration is so challenging), but bone repair needs to take angiogenesis into account.^[^ ^166^, ^167^ ^]^ The use of vascular endothelial growth factor (VEGF) within scaffolds has been intensely studied^[^ ^166^ ^]^ and may be an option for supporting the recreation of microvascular complexity within carbon‐based scaffolds. Besides, considering that growth factors are expensive and degrade rapidly, other strategies may be applied, including the use of fugitive inks, the employment of sandwiched constructs, the use of hollow fibers, or the combination of different additive manufacturing technologies operating across scales and employing varied materials.^[^ ^165^ ^]^

In the area of carbon‐based materials, the utilization of 3D printed graphene and CNTs,^[^ ^61^, ^140^ ^]^ with hollow micro‐tubular structures, might synergize with endothelial cells and VEGF and support blood vessel growth. In the area of muscular repair, carbon nanotubes have been^[^ ^168^ ^]^ found useful as VEGF carriers, so several types of CNTs‐based composites may benefit from this carry and delivery ability. 4D printing principles have been also proposed for promoting vascularization in tissue engineered constructs.^[^ ^169^ ^]^ These 4D principles may also be almost directly translated to lattice carbon structures, as further discussed in Section 6 when dealing with “living carbon,” which may constitute another strategy for achieving vascular complexity in carbon‐based scaffolds. Another interesting approach may be based on the pyrolysis of biological templates, whose intricate 3D structures, combining macro‐ and micro‐pores and showing interesting surface topographies, have been proposed for bone tissue engineering.^[^ ^170^ ^]^ Exploring increasing numbers of biological materials with hierarchical structures, including wood,^[^ ^115^ ^]^ sea plants,^[^ ^170^ ^]^ and leaves,^[^ ^171^, ^172^ ^]^ among others, and converting them into multi‐scale carbon scaffolds, may further transform tissue engineering through bioinspiration principles.

### 5.3 From the Lab to Industrialized Production and Mass Customization

Once the aforementioned key challenges are solved, a conversion from the lab to an industrial production of carbon‐based scaffolds will be also required, to achieve real clinical impact. Serial production and mass‐customization strategies may be compatible, and even synergetic, considering the variability of carbon‐based materials and the myriad of synthesis and processing techniques applicable, and the requirements for functionally graded scaffolds capable of recreating the complex cellular microenvironments in articular pathologies. Still, for supporting this transition, additional efforts are needed for creating the carbon‐based building blocks for tissue engineering, as a set of standardized components and processes that may reformulate the field of regenerative medicine.

For instance, the use of building blocks has proven already extremely effective for the industrial success and societal impact of extremely varied research creations and fields, from micro/nanoelectronics^[^ ^173^ ^]^ and microfluidics,^[^ ^174^ ^]^ to synthetic biology.^[^ ^175^ ^]^ The need for standardization in cell and tissue engineering has been previously highlighted^[^ ^176^ ^]^ and is also linked to the definition of methods and protocols to address gender‐related aspects, and are intimately connected to the safety and regulatory issues described in the following subsections. Such standardization may well start with agreements and precise descriptions of the actual building blocks,^[^ ^177^ ^]^ with lexical semantic approaches to terminology,^[^ ^178^ ^]^ and with open‐source libraries of standardized elements that researchers may use for increasing repeatability and comparability of results.^[^ ^179^ ^]^ Subsequently, these normalization advances may specialize into updated regulations and specific standards (see Section 5.5).

### 5.4 Addressing Gender‐Related Aspects in Articular Repair

Progressing toward standardized testing procedures that may support the expansion of carbon‐based materials in tissue engineering and regenerative medicine in considering gender‐related aspects, is vital, especially for articular pathologies. In fact, women suffer from osteopenia, osteoporosis and osteoarthritis more so than men, and at earlier ages^[^ ^180^, ^181^ ^]^ too. However, most studies in the tissue engineering field still do not plan experiments to explore gender‐specific issues, and most in vivo animal models used in biomedical research are male, for simplicity, and to avoid hormonal variations.^[^ ^182^ ^]^ Planning and developing studies for adequately addressing gender specific aspects in articular tissue engineering may involve the use of bioreactors with different levels of biomechanical stimuli, the incorporation of hormones to the cultured scaffolds, cells and tissues, or the support of animal populations that can evidence gender differences.^[^ ^183^ ^]^ To the best knowledge of the authors, no research study dealing with innovative carbon‐based materials for tissue engineering has also focused on gender differences, which should be corrected in the near future.

### 5.5 Safety and Regulatory Issues

Before reaching patients, carbon‐based scaffolds and carbon‐based cell therapies should fulfill important regulatory requirements and overcome comprehensive standardized trials. In the particular case of the European Union, carbon‐based biomaterials or carbon‐based scaffolds, to be used as advanced implants, must comply with the EU Medical Device Regulation 2017/745 and are classified as Class III medical devices, both in the case of mass‐produced and customized solutions.^[^ ^184^ ^]^ Accordingly, several standards have proven useful for demonstrating conformity, such as ISO 10993 for evaluating biocompatibility, ISO 13485 for quality management in medical device manufacturing, ISO 14917 for risk management techniques applied to medical devices, and ISO 14155 for planning clinical investigation with human subjects, among others. If the scaffolds involve cells and tissues, the situation is even more challenging, as they should be considered advanced therapy medicinal products, under EC Regulation 1394/2007. Here, together with the previously listed standards, additional good practices for testing and manufacturing should be promoted. ISO 14644 should be considered for cleanrooms, and ISO 20387 for biobanks, while ISO 18362, ISO 22442, and ISO 13022 should be taken into account for controlling risks in cell and tissue related therapies. Additionally, multifaceted ethical issues arise when dealing with enhancing life and creating living materials, as further considered in Section 6. In consequence, it is important for researchers to understand the technology transfer routes for these advanced biomaterials, and the legal aspects that already scaffold the tissue engineering field. Joining forces with regulatory experts in tissue engineering projects is extremely good advice.

## 6 Perspective: Engineering Carbon‐Based Living Materials

The increasing number of applications of carbon‐based materials for supporting cell culture processes, and their use as tissue engineering scaffolds, together with parallel progress in the emergent fields of engineered living materials (ELMs)^[^ ^185^, ^186^, ^187^ ^]^ and machines,^[^ ^188^, ^189^ ^]^ make us envision a promising future for a new research topic: the engineering of living carbon. Without question, carbon is the most important element for life on our planet. The carbon‐hydrogen bond is fundamental in organic chemistry and all known biological systems on Earth. Although some noncarbon biochemistries have been proposed,^[^ ^190^, ^191^ ^]^ their versatility is extremely limited, as compared with the benefits provided by the remarkable and exotic properties of the carbon atom, and derived carbon‐based materials. However, the emerging field of engineered living materials has not yet systematically focused on exploring and developing hybrid living materials that employ carbon allotropes, as essential constituents together with eukaryotic and prokaryotic cells. Interestingly, carbon‐based materials have been used for interacting at a cellular level since the dawn of tissue engineering, as already mentioned, and carbon micro/nanofabrication technologies have recently promoted various industrial revolutions. Nevertheless, most existing engineered living materials still employ only cultured cells, or cells combined with hydrogels and polymers,^[^ ^185^, ^186^, ^187^, ^192^, ^193^ ^]^ possibly as a consequence of recent progresses in bioprinting,^[^ ^194^, ^195^ ^]^ a technology that provides 3D versatility but still lacks precision and does not employ high‐performance materials.

In the authors' opinion, hybrid living carbon materials (HLMCs) may outperform pioneering examples of biological ELMs entirely synthesized with living cells, and of hybrid living materials made of cells and scaffolding structures. According to recent research, the former (scaffold‐less option) may lack structural integrity for high‐performance engineering solutions,^[^ ^196^ ^]^ like those needed in musculoskeletal tissue engineering, or in articular repair, for which large numbers of cells are required. On the other hand, the latter normally rely on hydrogels and polymerics,^[^ ^195^, ^197^ ^]^ whose biocompatibility and biomechanical properties are often sub‐optimal.

Carbon‐based materials, as scaffolding solutions for biohybrid structures, can be biomechanically fine‐tuned to cover the whole spectrum of interest for the replacement and regeneration of human tissues, and their biocompatibility is also noteworthy, as presented and discussed in previous Sections 2– 5. Arguably they can be engineered as the most promising cell niches, both for sustaining multicellular eukaryotic colonies^[^ ^198^ ^]^ and for hosting most of the prokaryotic cell types,^[^ ^199^ ^]^ which paves the way for advanced biomedical applications. Indeed, recent research has put forward the interest in using prokaryotes for controlling eukaryotes in engineered living systems,^[^ ^200^ ^]^ and carbon‐based solutions may be applied to advancing in this direction. Besides, the electrical conductivity of carbon‐based materials may enable self‐sensing and signaling within these novel HLMCs and support their autonomous operation and environmental adaptability, while also benefiting from carbon's chemical inertness and transparency to imaging technologies for monitoring purposes. Furthermore, the viability of 4D printing with carbon structures, demonstrated by our team, may support the engineering of shape‐morphing living carbon. Taking benefit from the aforementioned possibilities, some of the most relevant features proposed for living materials and living machines, like self‐assembly, shape‐morphing, self‐sensing, and even autonomous operation and healing, may be achieved with innovative HLMCs for the benefit of articular tissue engineering, as explained in the following subsections. In any case, although we are experiencing the birth of a new generation of living carbon materials and devices governed by cells, outstanding research efforts are still needed to truly bring HLMCs to life.

### 6.1 Self‐Assembly toward Large‐Scale Defect Reconstruction

The regeneration of large‐scale defects is one of the unsolved challenges in tissue engineering, as scaffolds of several cm^3^ require extremely large cell colonies and culture durations for cell expansion, gene expression and differentiation, and demand invasive surgeries leading to extended recovery processes. Large musculoskeletal and articular defects may benefit from the use of smaller scaffolding units, designed for self‐assembly, which could be easily delivered to the defect, as independent and well‐colonized units, and then combined into stable 3D autonomously assembled constructs. Self‐assembled scaffold‐based and scaffold‐free solutions for tissue engineering have been previously reviewed.^[^ ^201^ ^]^ Considering that 3D carbon‐based scaffolds can be achieved through additive manufacturing and consequent pyrolysis, the comprehensive experience of 3D printed designs for self‐assembly^[^ ^202^, ^203^ ^]^ may be almost directly transferred to the carbon field. Working with porous carbon “LEGO‐like” units or building bricks can further increase the versatility of carbon‐based materials for articular tissue engineering and support the transition toward living carbon.

### 6.2 Smart Responses: Shape‐Morphing and Autonomous Operation

**Figure 8:**
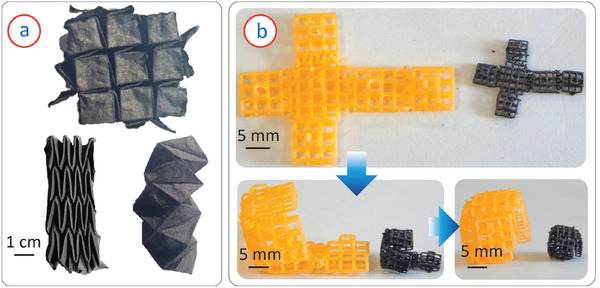
a) Examples of 3D carbon origami structures and b) sequential configurations of a shape‐morphing mechanism before and after carbonization, showing the retention of shape‐morphing capabilities after carbonization.

Designing for self‐assembly and shape‐morphing is central to the promotion of 4D printing processes, through which tissue engineering is being transformed. Indeed, the possibility of manufacturing scaffolding structures that undergo geometrical evolutions, during implantation, healing, and a patient's life, is a research breakthrough in tissue engineering.^[^ ^204^, ^205^ ^]^ As happens with self‐assembly, the experiences with design strategies for 4D printing and shape‐memory polymers^[^ ^203^, ^206^, ^207^ ^]^ may be straightforwardly transferred to carbon‐based materials, to achieve shape‐morphing carbon scaffolds. In articular tissue engineering, these smart responses may synergize with other minimally invasive surgical procedures and help to deliver the correct epigenetic stimuli during the healing and regeneration processes, as well as grow with the patient, which is fundamental for pediatric tissue engineering. Our pioneering work with carbonaceous origami structures (**Figure** **8**a ^[^ ^132^, ^208^ ^]^) and preliminary research on printed carbon mechanisms (see Figure 8b) provide initial results in this area of shape‐morphing or 4D printed carbon. These shape‐morphing scaffolds, once multi‐cellular cultures are employed for inducing controlled geometrical changes, may support the creation of successful living carbon‐based materials and biological micromachines with carbon chassis for a wide set of foreseeable industrial applications.

### 6.3 Smart Responses: Self‐Sensing Carbon Structures

Resourceful smart responses imply both actuating and sensing abilities. Self‐sensing scaffolds and biohybrid structures have been explored for tissue engineering, as a way for monitoring the state of the regeneration process and the structural integrity of the scaffolding elements and associated tissues. The monitoring of cellular activity using impedance measurements^[^ ^209^ ^]^ has been demonstrated, and potentials of self‐sensing biomaterials and scaffolds for cardiac^[^ ^210^ ^]^ and bone^[^ ^211^ ^]^ tissue engineering have also been studied and described. Carbon‐based materials and derived scaffolds can be seen as smart materials and structures for their intrinsic self‐sensing ability, thanks to their electrical conductivity, and to related variations due to eventual material scavenging, or to the propagation of cracks. Experiments with self‐sensing carbon‐based tissue engineering scaffolds have been reported: natural wood, delignified, carbonized, and backfilled with epoxy has been employed to achieve conductive scaffolds for electronic applications;^[^ ^212^ ^]^ carbon nanotube polymer composites have been used to monitor cell activity;^[^ ^209^ ^]^ and the already described properties of carbon dots can also support the creation of self‐sensing carbon structures.

Once the self‐sensing properties of carbon structures are combined with the precise actuation achievable using biohybrid cell‐based actuators for microsystems^[^ ^213^ ^]^ and with the additional possibilities provided by shape‐morphing carbon scaffolds and micromechanisms, the new field of carbon‐based living materials and machines is expected to emerge and lead to applications beyond the biomedical field, possibly including micropositioning in production systems, smart actuators for resource limited and harsh environments, and energy production.

### 6.4 Self‐Healing Properties and Self‐Sufficient Production

Living systems still outperform synthetic biology in many ways, especially regarding their self‐healing capabilities. Therefore, the development of synthetic self‐healing tissue engineering scaffolds has been also a matter of intense research for years, normally involving hydrogels,^[^ ^214^ ^]^ whose soft and elastic behavior present potential for cartilage repair and regeneration, as the self‐healing properties of living cartilage are limited. Carbon‐based scaffolding structures with self‐healing ability have been also pursued. For example, the self‐healing ability of graphene and graphene‐based composites have been studied and applications in varied areas, including tissue engineering, smart medical devices, and structures with self‐sensing properties, have been proposed.^[^ ^215^, ^216^, ^217^, ^218^ ^]^ Varied polymer‐carbon nanocomposite systems with self‐healing properties have been also developed, in which carbon fibers and nanotubes have been crucial components.^[^ ^219^, ^220^, ^221^, ^222^ ^]^

### 6.5 Ethical and Social Aspects

Carbon‐based tissue engineering scaffolds, in their expected transition toward engineered living carbon, will enter the realm of synthetic biology. In consequence, not only the fulfillment of ISO 10993 requirements and compliance (in Europe) with the MDR 2017/745, or with other applicable regulations, will be necessary, but additional yet fundamental ethical and social aspects will arise. Indeed, ethical issues in the synthetic biology and living materials fields–bioethics–are getting increased attention,^[^ ^223^, ^224^, ^225^ ^]^ as for ethical concerns of nanotechnology and nanomaterials research–nanoethics.^[^ ^226^, ^227^ ^]^ In the specific area of tissue engineering (acting at the interface between synthetic biology, living materials, and nanotechnology) ethical considerations linked to cell sources, the use of human embryonic stem cells, therapeutic cloning, maintenance of records, and respecting privacy, to cite a few, have been highlighted.^[^ ^228^, ^229^ ^]^ The incorporation of carbon‐based materials and nanocomposites into the portfolio of tissue engineering constructive elements should also take account of potential risks and analyze risk‐benefit ratios, as highly original studies focusing on the ethics of carbon nanotubes^[^ ^88^ ^]^ and carbon graphene research^[^ ^230^, ^231^ ^]^ have proposed. Thinking about a future of living materials and machines, in which carbon‐based materials may play a highly relevant role, training a new generation of researchers capable of acting at the interface between biology and technology seems to be a necessary approach. In this direction, it is necessary to design programmes of study capable of merging an in‐depth focus on scientific‐technological knowledge and technical abilities, with a comprehensive understanding of social and ethical implications, and a promotion of soft‐skills, for optimally deploying the techniques of Industry 4.0 and 5.0 for the benefit of Society 5.0, as proposed in the Engineering Education 5.0 paradigm.^[^ ^232^ ^]^ Following recommendations and methods from pioneering examples, dealing with integrating social and ethical implications within nanotechnology education,^[^ ^233^, ^234^ ^]^ is advisable.

**Table 1:** Summary of carbon materials used in articular tissue engineering: focus on available clinical data, biocompatibility and toxicity

Type of carbon	Brief description of scaffolding structure	Objective articular tissue	Validation and available clinical data	Described biocompatibility issues	Reference
	Fiber bundles as innovative prostheses	Ligament and tendon	Long‐term ACL and Achiles tendon repair	Adequate tolerance and in vivo performance	[77]
	Carbon fibers coated with gelatin	Ligament	Long‐term in vivo studies show collagen formation	Mild inflammation. Brittleness and possible debris	[78]
	Carbon fibers with joint capsule or lyophilized dura	Ligament	ACL reconstruction (37 patients after 8 years)	Unacceptable long‐term increase of osteoarthrosis	[10]
	Carbon fiber pads	Cartilage	Long term repair of condyles	Successful results in knee condyle after 5.8 years	[71]
	Woven carbon fiber pads	Cartilage	in vivo studies in rabbit models for 6 weeks	Successful healing and mechanical endurance	[72]
	Carbon fiber composite bone plates	Bone	in vivo studies in 40 forearm fractures	All fractures united, 67% showed remodeling within 6 months, 5 showed an unexpected reaction	[73]
	Carbon fiber/flax/epoxy sandwich	Bone	In vitro fatigue testing	Potential candidate with adequate fatigue properties	[79]
	Carbon fiber web with bone morphogenic protein	Bone	in vivo studies in murine models	High bone‐tissue compatibility	[80]
Carbon fibers and meshes	Electrospun carbon nanofibers	Bone	Varied In vitro and in vivo studies are reviewed	Further studies are advised	[81]
CNTs	Block structure made of carbon nanotubes	Bone	in vivo studies in mouse	Bone formation is verified, which shows potentials	[82]
	Bioprinted CNT‐reinforced scaffolds	Bone and cartilage	Varied In vitro and in vivo studies are reviewed	Toxicity and means for its limitation are discussed	[83]
	2D CNT sheets and 3D CNT textiles	Cartilage	In vitro studies	Cartilage growth is verified, which shows potentials	[84]
	CNT‐based biomaterials	Varied tissues	Varied In vitro and in vivo studies are reviewed	Biocompatibility and carcinogenicity are discussed	[56]
Graphene and its derivatives	3D printed graphene composites	Soft tissues	In vitro studies with hMSCs	Promising biocompatibility along 30 days	[95]
	Graphene combined with biomaterials	Bone	Varied In vitro studies are reviewed	Enhanced osteogenic responses are found	[98]
	Graphene based biomaterials	Bone	Varied In vitro and in vivo studies are reviewed	Biocompatibility and its enhancement are discussed	[48]
	Graphene oxide flakes in hydrogels	Cartilage	In vitro studies with hMSCs	Efficient delivery of biofactors and tissue growth	[99]
	Polymer scaffolds with graphene and CNTs	Bone and cartilage	Varied In vitro studies are reviewed	Biomimetic performance, acceptable biocompatible	[101]
	Graphene‐polymer composites	Ligament and tendon	Varied In vitro and in vivo studies are reviewed	Promising biocompatibility of graphene surfaces	[102]
Carbon dots	rBMSCs labeled with carbon dots	Bone	In vitro studies with rBMSCs	Promotion of osteogenic differentiation	[109]
	Carbon dots in electrospun nanofiber mats	Varied tissues	In vitro studies	Low cytotoxicity, enhanced cell proliferation	[110]
	Carbon dots in biomedical applications	Varied tissues	Varied In vitro studies are reviewed	Low toxicity compared to other quantum dots	[111]
Glassy carbon	Reticulated vitreous carbon scaffolds	Bone	In vitro (rMSCs) and in vivo (rabbit) studies	Successful repairs, minor concerns, extremely detailed discussion about biocompatibility	[112]
	Reticulated vitreous carbon foams	Bone	In vitro studies: cytotoxicity and cell adhesion assays	Suggest appropriate results for biomed applications	[113]
	Bioglass coated glassy carbon scaffolds	Bone	In vitro studies	Potential for bone formation is verified	[114]
	Design‐controlled glassy carbon scaffolds	Varied tissues	In vitro studies	Remarkable cell adhesion In vitro	[116]
	Multi‐scale glassy carbon scaffolds	Varied tissues	In vitro studies	Remarkable cell adhesion In vitro	[117]
Nano‐diamond and DLC	DLC coated tissue engineering scaffolds	Varied tissues	In vitro studies with hMSCs	Remarkable cell adhesion In vitro	[118]
	Substrates with NDs and NDs‐polymer composites	Bone	Varied In vitro studies are reviewed	Better cell growth and osteogenic differentiation	[119]
	DLC coated polymeric sheets	Varied tissues	In vitro studies with h‐umbilical endothelial cells	Promising biocompatibility of DLC coatings	[121]
	DLC coated nitrocarburized steel scaffolds	Bone	Piting and corrosion tests	Improved biocompatibility of metallic implants	[122]

## Conflict of Interest

The authors declare no conflict of interest.
